# Valsartan alleviates the blood–brain barrier dysfunction in db/db diabetic mice

**DOI:** 10.1080/21655979.2021.1981799

**Published:** 2021-10-26

**Authors:** Longxue Cai, Wenfeng Li, Renqing Zeng, Zuohong Cao, Qicai Guo, Qi Huang, Xianfa Liu

**Affiliations:** a Department of Emergency, First Affiliated Hospital of Gannan Medical University, Ganzhou City, Jiangxi Province, China; b Department of Cardiology, Ganzhou People’s Hospital, Ganzhou City, Jiangxi Province, China

**Keywords:** Valsartan, blood-brain barrier (BBB), db/db mice, human brain microvascular endothelial cells (HBMVECs), cAMP-responsive element-binding protein (CREB)

## Abstract

Type 2 diabetes (T2D)-related neurological complication is the risk factor for neurodegenerative disorders. The pathological changes from T2D-caused blood–brain barrier (BBB) dysfunction plays a critical role in developing neurodegeneration. The hyper-activation of the Angiotensin II type 1 receptor (AT1R) in the brain is associated with neurovascular impairment. The AT1R antagonist Valsartan is commonly prescribed to control high blood pressure, heart failure, and diabetic kidney diseases. In this study, we investigated the beneficial effects of Valsartan in db/db diabetic mice and isolated brain endothelial cells. We showed that 2 weeks of Valsartan administration (30 mg/Kg body weight) mitigated the increased permeability of the brain-blood barrier and the reduction of gap junction proteins VE-Cadherin and Claudin 2. In human brain microvascular cells (HBMVECs), we found that Valsartan treatment ameliorated high glucose-induced hyperpermeability by measuring Dextran uptake and transendothelial electrical resistance (TEER). Furthermore, Valsartan treatment recovered high glucose-repressed endothelial VE-Cadherin and Claudin 2 expression. Moreover, Valsartan significantly suppressed the expressions of pro-inflammatory cytokines such as macrophage chemoattractant protein-1 (MCP-1) and interleukin-6 (IL-6) against high glucose. Mechanistically, Valsartan ameliorated high glucose-repressed endothelial cAMP-responsive element-binding protein (CREB) signaling activation. The blockage of CREB activation by PKA inhibitor H89 abolished the action of Valsartan, suggesting its dependence on CREB signaling. In conclusion, Valsartan shows a neuroprotective effect in diabetic mice by ameliorating BBB dysfunction. These effects of Valsartan require cellular CREB signaling in brain endothelial cells.

## 1. Introduction

The prevalence of type 2 diabetes (T2D) has become one of the major health threats. It has been estimated that about half of T2D patients will have T2D-related complications [1]. Complications of diabetic neuropathy affect many aspects of quality of life. As a result, T2D is now a major risk factor for many neurological disorders such as stroke, dementia, and Alzheimer’s disease (AD) [2], which have high morbidity and high mortality. Pathological studies have shown that the T2D-caused blood–brain barrier (BBB) dysfunction plays a critical role in these neurological complications [3]. The BBB serves as a highly selective border for circulating blood and other extracellular fluid in the brain and is composed of microvascular endothelial cells, astrocytes, pericytes, and a capillary basement membrane. Endothelial cells connect and form tight junctions to provide the physical wall for fluid circulation in the brain [4]. The tight control of the integrity of the BBB is fundamental to brain health, and the development of a drug targeting BBB damage in T2D is clinically significant. Also, the BBB has also been important for drug development as its permeabilization is essential for the treatment of brain disorders. There are several tight junction proteins on the brain capillary walls, such as Claudins, Occludins, and VE-cadherin. These transmembrane molecules are linked to the cytoskeletal molecules, and their being intact is crucial to the function of the BBB [5]. The regulation of the BBB is a complex process with unknown mechanisms [6,7].

The renin-angiotensin system (RAS) is best known for its modulation of essential hypertension. Recently, it has been shown that renin-angiotensin system regulators have a pleiotropic role in the central nervous system (CNS), independent of their regulation of hypertension. We are now aware that the brain is susceptible to Angiotensin II (Ang-II), the major peptide in the RAS system. Ang II acts on its receptors (AT1R and AT2R) to regulate various physiological effects [8]. AT1Rs are G-protein coupled receptors (GPCRs) expressed in different brain cells. Hyperactivation of AT1R signaling in the brain is associated with oxidative stress, apoptosis, and neuroinflammation [9]. Regions of the brain outside the BBB, such as the circumventricular organs, may act as classic targets of endocrine Ang-II. Numerous studies have demonstrated solid neuroprotective effects by blocking AT1R in these brain disorders. Hence, inhibition of AT1R signals is beneficial in neuroprotection and cognitive improvement [10]. The AT1R antagonists (ARBs) are widely used as drugs for hypertension and heart failure, as well as diabetic nephropathy, but their effect on brain disorders is not well investigated. Clinical studies have shown that the administration of ARBs has benefits in preventing stroke [11] and dementia [12]. A systematic comparison study suggests that several types of ARBs can penetrate the BBB and have an impact on brain tissue, including Valsartan [13]. A series of animal experiments show that ARBs indeed have the ameliorative roles in improving cognitive impairment and memory loss in the animal models of Parkinson’s disease [14] and Alzheimer’s disease [15]. Valsartan is commonly prescribed in the clinical care of hypertension and heart problems, but its effect on brain disorders remains largely unknown. In this study, we tested the role of Valsartan on the brain tissues in diabetic mice and investigated its pharmacological effect in isolated brain vascular endothelial cells.

## 2. Materials and methods

### 2.1. Animals and experiments

About 8–12 weeks old age-matched male leptin receptor-deficient mice (db/db, #000642) or heterozygous mice (db/+) were purchased from Jackson Laboratories (Bar Harbor, USA). All *in vivo* experiments were performed following animal protocols approved by the ethical committee of Gannan Medical University. All mice were housed in a 12-h light/12-h dark cycle with free access to food and water. ARB drug Valsartan (#SML0142) was purchased from Sigma-Aldrich (St. Louis, USA). The animals were allocated to three groups: the heterozygous db/+ control, db/db with Valsartan, db/db as the placebo. Valsartan (or the placebo) was administered in db/db mice at a dose of 30 mg/kg by intraperitoneal injection for 2 weeks.

### 2.2 Brain permeability

The blood–brain barrier (BBB) permeability was measured using sodium fluorescein (NaFl) permeability assay as described before [16]. For the NaFl method, mice were intraperitoneally injected with NaFl (200 mg/kg) on day 14 of Valsartan administration. The animals were then sacrificed for transcardial perfusion with phosphate-buffered saline (PBS) under anesthesia. The cortex sections were dissected to extract NaFl using 30% trichloroacetic acid method. NaFl fluorescence was quantified using a fluorescence microplate reader (BioTek, USA).

### 2.3. Real-time PCR

Total RNA was extracted from dissected brain or cultured cells with Trizol reagent (Thermo Fisher Scientific, USA) as described by the product’s manual. The purity and concentration of RNA were monitored with Nanodrop 2000 (Thermo Fisher Scientific, USA). To synthesize the cDNA, 1 μg RNA was used for reverse transcript RNA with a commercial one-step cDNA synthesis kit (Takara Bio, Japan). Then, the cDNA product was diluted with ddH_2_O to 200 µl, and 1 µl diluent was used for the real-time PCR reaction with SYBR Green Master Mix (Roche, Switzerland) on an ABI 7500 Real-Time PCR platform. The relative expression of the genes was calculated using the 2^−ΔΔCt^ method and normalized to glyceraldehyde-3-phosphate dehydrogenase (GAPDH).

### 2.4. Immunostaining

Mice were sacrificed and perfused by chilled saline for 4 minutes and followed by 4% paraformaldehyde for 8 minutes. The whole-brain tissue was dissected and soaked in 30% sucrose solution overnight. Then, the tissues were frozen and sectioned on a Cryostat (Leica, USA) to get 8 µm thin sections. The cortex sections were permeabilized with 0.3% Triton X-100 in PBS for 10 minutes, then blocked in PBS with 10% horse serum for 1 h. After that, the sections were incubated with primary antibodies (VE-cadherin: 1: 100, Abcam #78,747; mouse Claudin 2, 1:100, #Abcam 9391) in a blocking buffer at 4°C overnight. Finally, the sections were incubated with anti-mouse secondary fluorescent antibodies (Thermo Scientific, USA) for 1 h and mounted with prolonged gold antifade media (Thermo Scientific, USA) at room temperature. The slides were visualized with a laser confocal microscope (Leica TSC STED).

### 2.5. Cell culture and treatment

Primary human brain microvascular endothelial cells (HBMECs) (#ACBRI376) were purchased from Cell Systems (Kirkland, USA). The cells were maintained in CSC complete medium from the vendor (#4Z0-500; Cell Systems) supplied with 10% fetal bovine serum (FBS) and growth factors. All the cell experiments were performed within 4–10 generations of cell passage. The cells were seeded with a density of 10,000 cells/cm^2^ and allowed to grow to the full confluence. To mimic high glucose exposure, the monolayer cells were exposed to CSC growth media supplemented with high glucose (30 mM), with growth media supplemented with normal glucose (5 mM) as control. To test the effect of Valsartan, the cells were also treated with 5 μM Valsartan. The concentration of Valsartan in this study was based on previous studies in endothelial cells [17,18] and tested in our experiment.

### 2.6. Endothelial permeability

The permeability of HBMVECs was measured using FITC-Dextran uptake. In brief, HBMVECs were grown on monolayer inserts of Transwell, the cells were incubated with FITC-Dextran containing media for 30 minutes. Media in the lower chamber were transferred to a 96-well plate. The FITC-Dextran fluorescence was quantified. Fluorescence values were normalized by the total protein amount based on the Bradford assay. The data presented a fold change.

### 2.7. Trans-endothelial electrical resistance

The trans-endothelial electrical resistance (TEER) was measured as described in the previous study [16]. In brief, HBMVECs were cultured on the inner surface of fibronectin-coated 24-well Transwell inserts (Corning) incomplete CSC media with normal (5 mM) glucose or 30 mM high glucose. At the full confluence, the inserts were transferred into the EndOhm-6 chamber (World Precision Instruments). Then, 0.1 M KCl was added to the chamber. The EndOhm cap was then inserted at the top of the chamber, and Transwell was connected to a connector cable and capped, and resistance was read by the EVOM resistance reader meter (World Precision Instruments). The Transwell containing 0.1 M KCl but without the cells was used as a blank control. The data were normalized to control and presented as fold change.

### 2.8. Western blot

The proteins were obtained either from mice cortex tissue or cultured cell monolayers. To extract the soluble protein from the tissue, the collected brain tissues were homogenized in radioimmunoprecipitation (RIPA) buffer. The whole-cell lysates were directly collected by lysing the cell in RIPA buffer. The protein concentration was measured using a BCA protein assay kit (Thermo Fisher Scientific, USA). The samples were denatured on the heating block for 5 minutes, and 20 µg protein was loaded on a 10% sodium SDS-PAGE gel for electrophoresis. The proteins on the gel were then transferred onto a PVDF blot (Millipore). The blot was incubated with the following primary antibodies overnight at 4°C and followed by secondary anti-rabbit or anti-mouse antibodies (Cell Signaling Technologies, 1:5,000) for 1 h at room temperature, and then reacted with chemiluminescence substrate (IRDye Li-COR, USA) for 5 minutes at room temperature. The following primary antibodies were used: AT1R (Abcam, ab124734, 1:500), VE-cadherin (Cell Signal, #2500, 1:1000), and claudin 2 (#48,120, 1:1000), p-CREB (Cell Signal, Phospho-CREB (Ser133) #9198, 1:1000), CREB (Cell Signal, #9197, 1:1000), and β-Actin (Cell Signal, #3700, 1:5000). Finally, the bands on the membrane were visualized and analyzed using the Odyssey near-infrared imaging system (Odyssey LI-COR, USA), and the dosimetry was analyzed using the Odyssey 2.1 software.

### 2.9 Statistical analysis

All the experimental data were expressed as mean ± standard error of the mean (S.E.M.). All the statistical test was performed by Prism 8 software (GraphPad). To compare the mean of the two groups, a two-tailed Student’s t-test was used. For the comparison of more than two groups, ANOVA followed by Bonferroni corrected post-hoc test was performed. A p-value <0.05 was determined to be statistically significant.

## 3 Results

To investigate the effect of the AT1R antagonist Valsartan, both diabetic mice and high glucose stress brain endothelial cells were employed. We administered db/db mice with Valsartan (30 mg/Kg body weight) for 2 weeks and examined its therapeutic effect on the mouse brain-blood barrier. In cultured human brain microvascular cells (HBMVECs), we showed the protective role of Valsartan in high glucose-elicited endothelial hyperpermeability. We also demonstrated the inhibitory effect of Valsartan on pro-inflammatory cytokines expression and the involvement of the endothelial CREB signaling pathway.

### 3.1 Elevated brain AT1R expression in db/db mice

**Figure 1.**
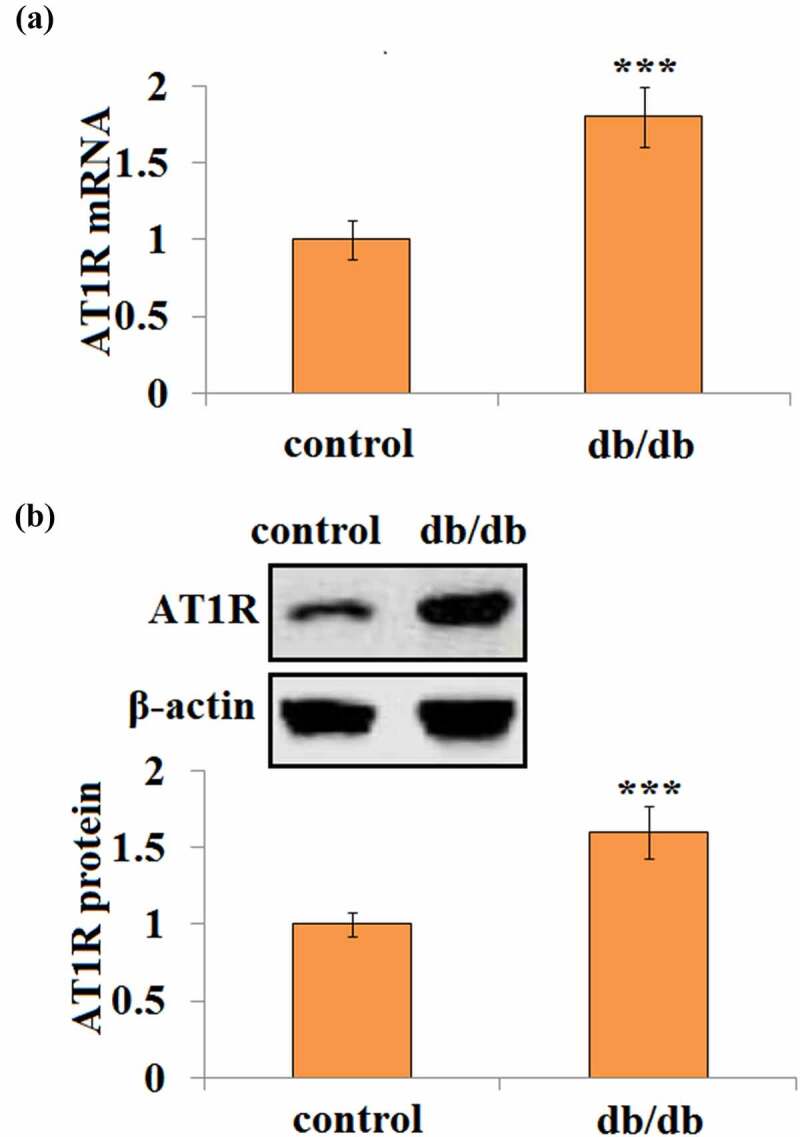
AT1R expression is elevated in the brain in db/db mice. (a) Quantification of AT1R mRNA in control and db/db mice; (b). Representative bands and quantification of AT1R protein in control and db/db mice(***, P < 0.005 vs. vehicle, N = 8)

First, the AT1R expression level was examined in the brain tissues of db/db and matched db/+ control mice. Interestingly, db/db mice had about 1.8-fold higher AT1R mRNA transcription than db/+ control mice (Figure 1a). Consistently, db/db mice showed a 1.6-fold higher AT1R protein level than control mice (Figure 1b).

### 3.2 Valsartan ameliorates brain permeability in db/db mice

**Figure 2.**
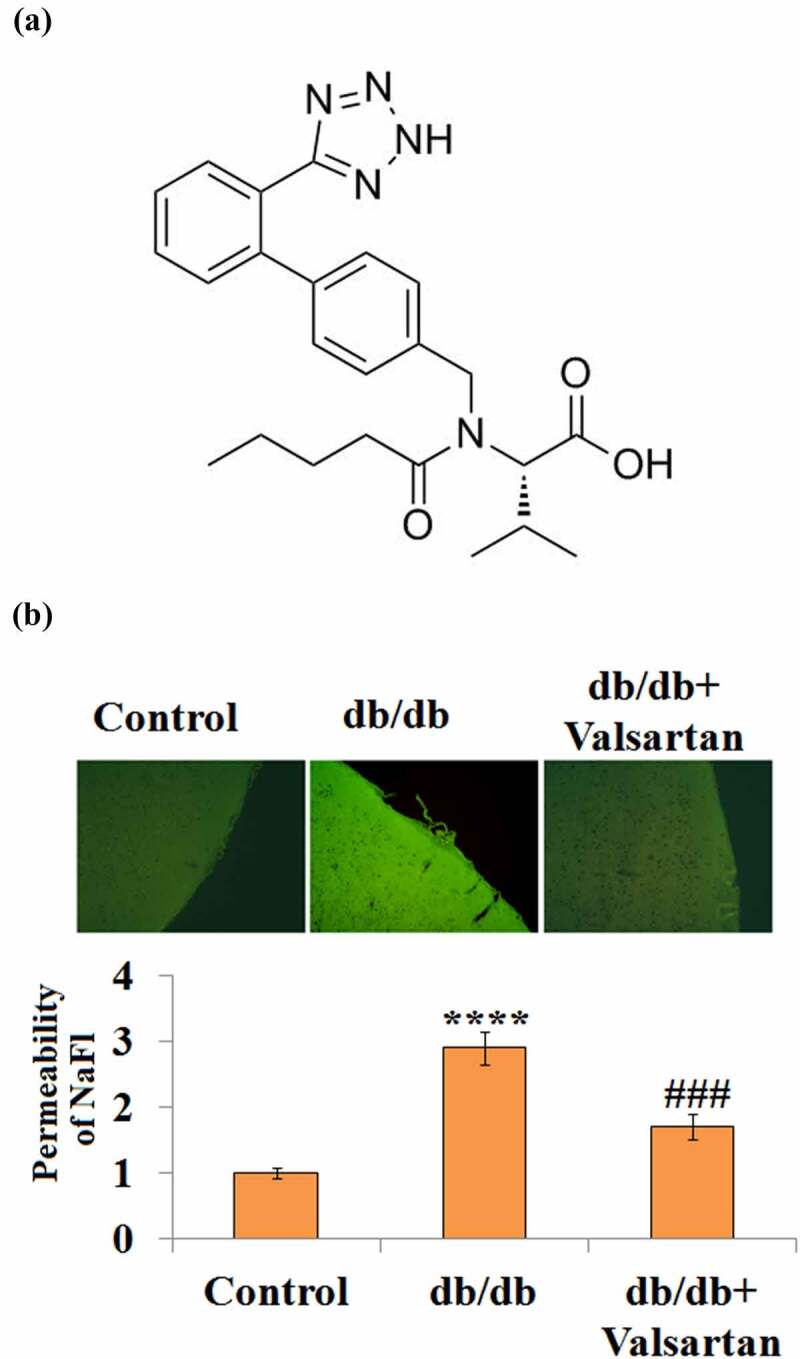
AT1R antagonist Valsartan attenuates diabetes-associated impairment of BBB integrity. (a). Molecular structure of Valsartan; (b). BBB permeability was measured using sodium fluorescein (NaFl)/fluorescein isothiocyanate (FITC)-Dextran permeability assay.(****, P < 0.001 vs. control, ###, P < 0.005 vs. db/db mice, N = 8)

Next, we sought to antagonize the AT1R level in db/db mice and examine the permeability. Valsartan is a monocarboxylic acid amide with a modified side-chain L-valine (Figure 2a), which acts to block the binding of Angiotensin II to AT1R in vascular tissue. Representative images of the BBB permeability of NaFl are shown in Figure 2b. Compared with the control group, placebo-treated db/db mice had about a 2.7-fold higher brain permeability; however, two-week Valsartan treatment in db/db mice had only 1.7-fold BBB permeability (Figure 2b), indicating Valsartan administration reduced BBB permeability.

### 3.3 Valsartan mitigates the expression of VE-cadherin and claudin 2 in db/db mice

**Figure 3.**
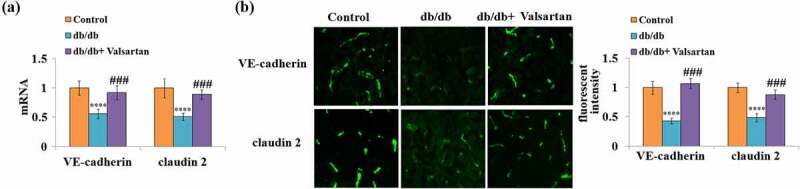
Valsartan restored the expression of VE-cadherin and claudin 2 in the brain of db/db mice. (a). Quantification of VE-cadherin and claudin 2 mRNA; (b). Fluorescent images of VE-cadherin and claudin 2 staining.(****, P < 0.001 vs. control, ###, P < 0.005 vs. db/db mice, N = 8)

To inspect the protective role of Valsartan administration on the BBB in db/db mice, the two key molecules of the BBB were examined in brain tissues. Valsartan administration showed robust mitigation of reduced brain VE-cadherin and claudin 2 mRNA expression in db/db mice (Figure 3a). Meanwhile, the immunostaining results confirmed that Valsartan treatment almost recovered reduced VE-cadherin and claudin 2 protein levels (Figure 3b).

### 3.4 Valsartan suppresses pro-inflammatory cytokines in cultured brain endothelial cells

**Figure 4.**
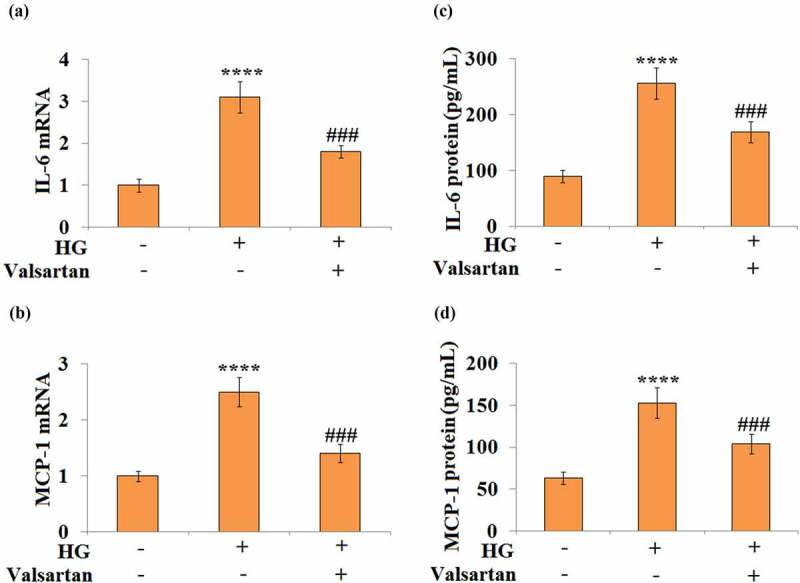
Valsartan suppressed pro-inflammatory mediators expression in high glucose (HG)-challenged human brain microvascular endothelial cells (HBMVECs). HBMVECs were incubated with 30 mM glucose or Valsartan (5 μM). (a). mRNA of IL-6; (b). mRNA of MCP-1; (c). IL-6 secretion; (d). MCP-1 secretion(****, P < 0.001 vs. control, ###, P < 0.005 vs. OGD/R, N = 5)

To mimic the hyperglycemia challenge in db/db mice, the high glucose-treated brain endothelial cell model was established to investigate the effect of Valsartan. As shown in Supplementary Figure 1a-B, exposure to high glucose significantly increased the expression of AT1R at both the mRNA and protein levels. In 30 mM glucose-treated HBMECs, both IL-6 and MCP-1 mRNA expression were induced. However, the addition of Valsartan showed significant suppression on both IL-6 and MCP-1 expression (Figures 4a-b). For their expression levels, our results show that glucose alone induced a several-fold increase of both IL-6 and MCP-1, but the addition of Valsartan suppresses the secretion of IL-6 and MCP-1 from HBMECs (Figures 4c-d).

### 3.5 Valsartan mitigates high glucose-induced hyperpermeability in brain endothelial cells

**Figure 5.**
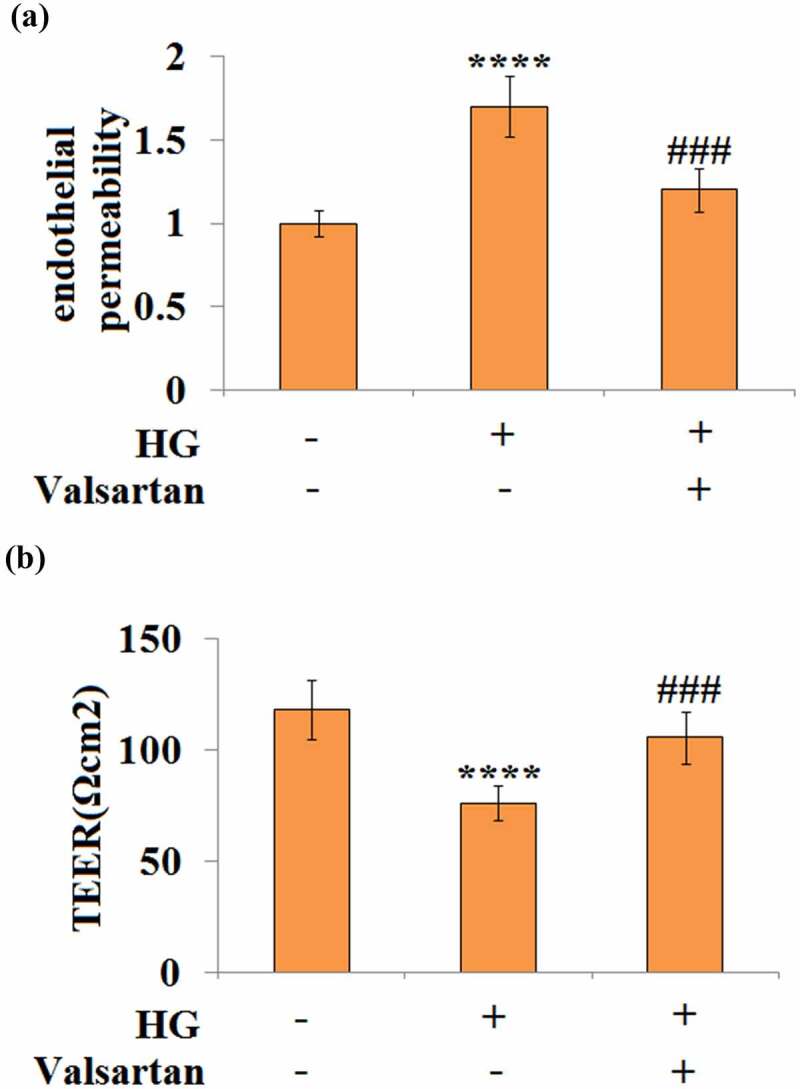
Valsartan ameliorated HG-induced enlargement of endothelial monolayer permeability in HBMVECs. HBMVECs were incubated with 30 mM glucose or Valsartan (5 μM). (a). Endothelial permeability; (b). The trans-endothelial electrical resistance (TEER).(****, P < 0.001 vs. control, ###, P < 0.005 vs. OGD/R, N = 5)

High glucose treatment is known to induce hyperpermeability in brain endothelial cells. Two different approaches were applied to determine the effect of Valsartan on endothelial permeability. By measuring the amount of Dextran uptake, our experiment showed that high glucose-induced about a 1.7-fold increase in permeability, but only about 1.2-fold permeability when Valsartan was added to the media (Figure 5a). For cellular trans-endothelial electrical resistance (TEER), our data show that high glucose reduced about one-third of TEER; however, the presence of Valsartan was able to ameliorate this suppressive effect, as it only reduced about one-tenth of TEER when Valsartan was present (Figure 5b).

### 3.6 Valsartan mitigates high glucose- reduced VE-cadherin and Claudin 2 expressions in brain endothelial cells

**Figure 6.**
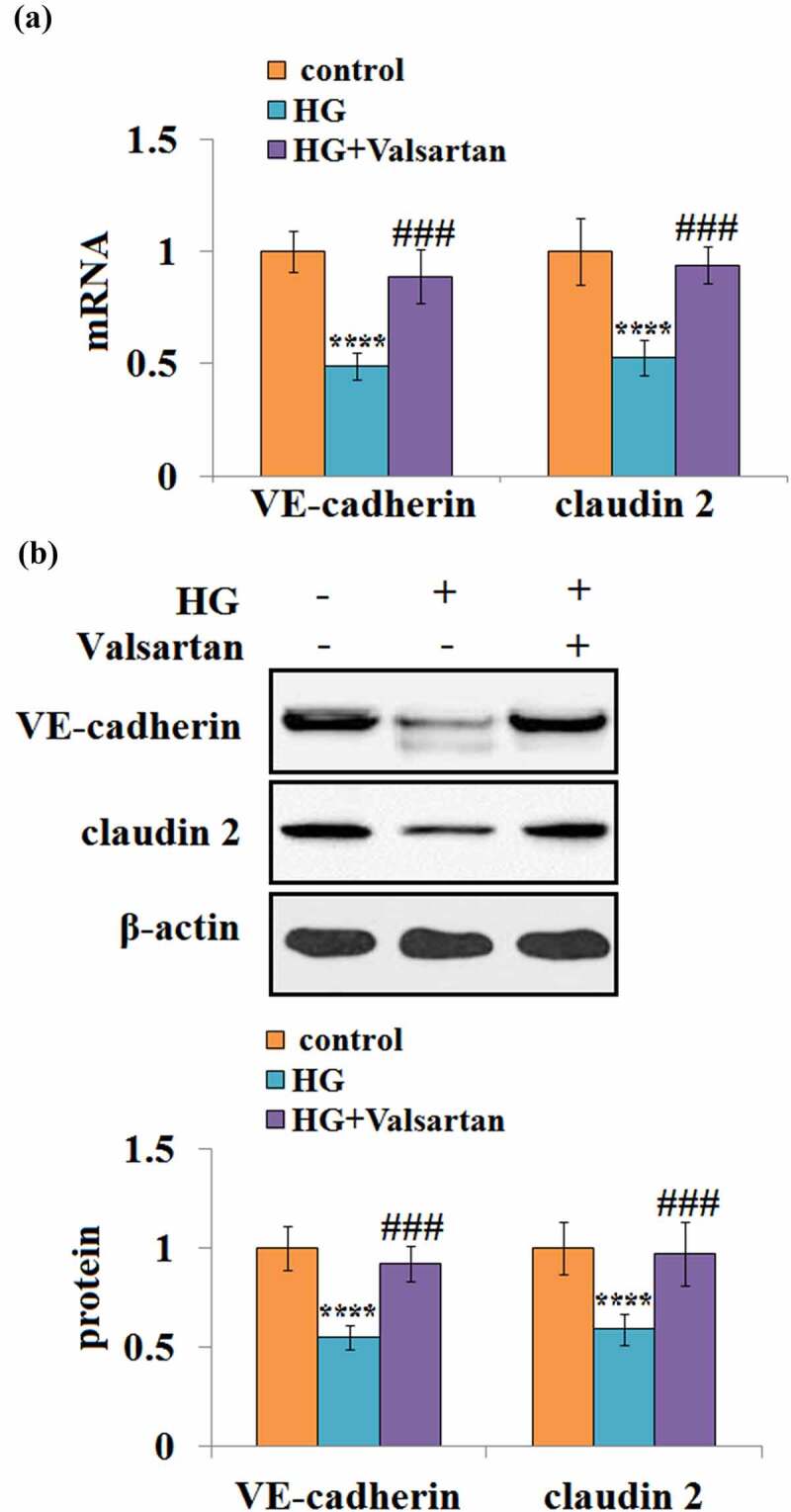
Valsartan restored VE-cadherin and claudin 2 expressions in HG-challenged HBMVECs. (a). VE-cadherin and claudin 2 mRNA expression; (b). VE-cadherin and claudin 2 protein expression as measured by western blot.(****, P < 0.001 vs. control, ###, P < 0.005 vs. OGD/R, N = 5–6)

Similarly, the effects of Valsartan on the VE-cadherin and Claudin 2 expression were examined. At the mRNA level, high glucose reduced about half of both VE-cadherin and Claudin 2. However, the presence of Valsartan greatly mitigated their suppression due to high glucose (Figure 6a). At the same time, the presence of Valsartan could attenuate the high glucose-suppressed protein levels of VE-cadherin and Claudin 2 (Figure 6b).

### 3.7 Valsartan ameliorates CREB inactivation

**Figure 7.**
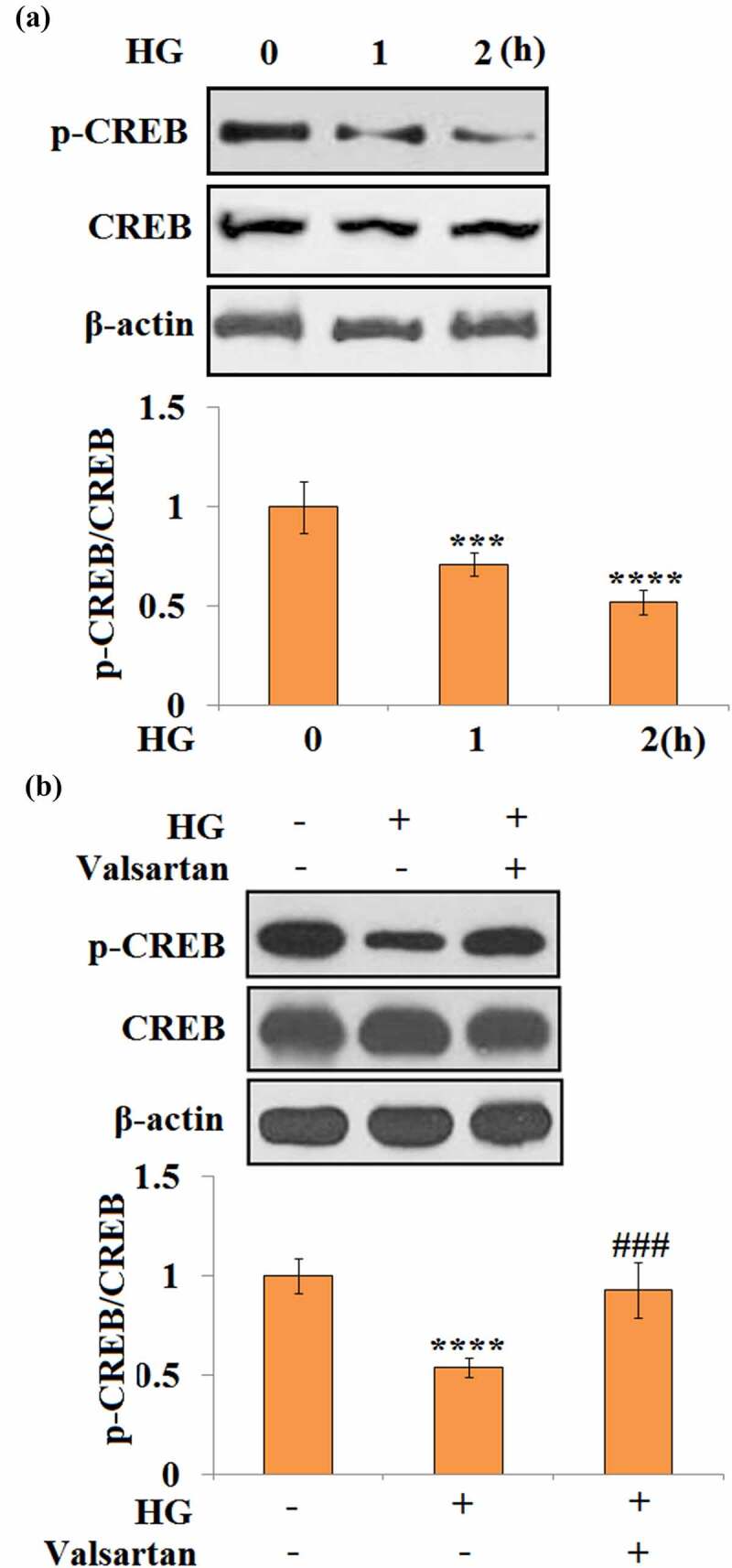
Valsartan ameliorated CREB inactivation in HG-challenged HBMVECs. (a). Cells were stimulated with HG for 1 and 2 hours. Phosphorylated (Ser 133) and total CREB were measured; (b). Cells were challenged with HG with or without Valsartan. Phosphorylated (Ser 133) and total CREB were measured. (****, P < 0.001 vs. control, ###, P < 0.005 vs. OGD/R, N = 5)

To understand the molecular mechanism of Valsartan, the CREB pathway was assessed in HBMECs. Phosphorylation of CREB at Ser 133 was evaluated. Notably, 1- and 2-h long treatments with high glucose reduced p-CREB to 71% and 52%, respectively (Figure 7a). Meanwhile, in the co-treatment experiment with high glucose and Valsartan, high glucose alone reduced p-CREB to 54%. However, it only caused a slight reduction of p-CREB, and 93% p-CREB was recovered when Valsartan was present (Figure 7b).

### 3.8 CREB activation mediates the effect of Valsartan

**Figure 8.**
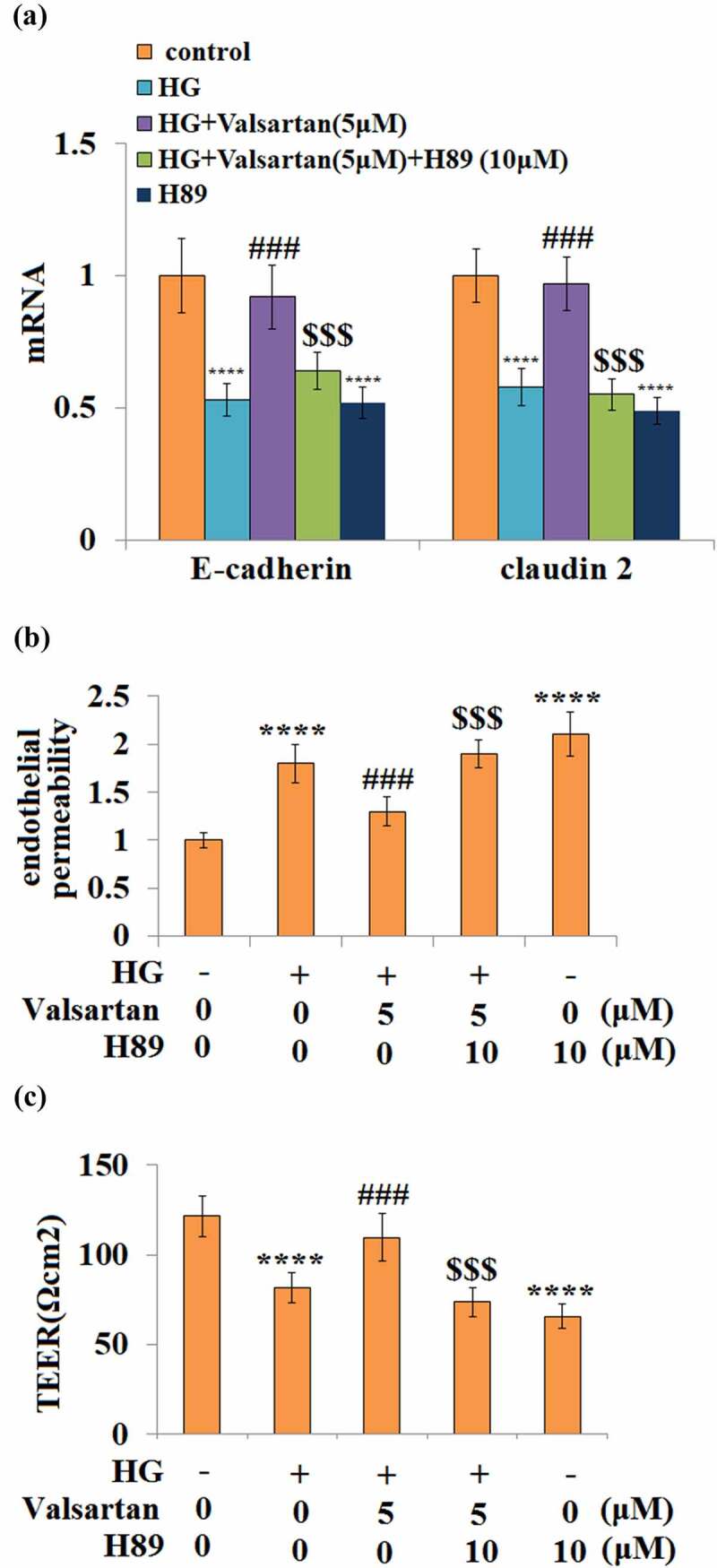
Blockage of CREB abolished the protective effects of Valsartan against HG-induced endothelial permeability and tight junction expression. Cells were treated with HG in the presence of Valsartan (5 μM) or H89 (10 μM) for 24 hours. (a). mRNA of E-cadherin and claudin 2; (b). Endothelial permeability; (B). The trans-endothelial electrical resistance (TEER) (****, P < 0.001 vs. control, ###, P < 0.005 vs. OGD/R; $$$, P < 0.005 vs. OGD/R+ Valsartan, N = 5)

Finally, the essential role of the CREB pathway in Valsartan-mediated protection was assessed using the PKA inhibitor H89. The co-treatment with Valsartan-ameliorated high glucose-reduced mRNA expressions of VE-cadherin and Claudin 2, however, the blockage of CREB activity by H-89 effectively abolished the ameliorative effect of Valsartan on the expressions of these two genes (Figure 8a). As shown before, the presence of Valsartan consistently ameliorated high glucose-induced hyperpermeability, but the blockage of CREB completely diminished its protection of endothelial permeability change (Figure 8b). Similarly, the blockage of CREB activity abolished Valsartan ameliorated TEER reduction due to high glucose (Figure 8c).

### 3.9. Knockdown of CREB abolished the protective effects of Valsartan

To further verify the protective effects of Valsartan, we knocked down the expression of CREB by infection with Ad-viral CREB shRNA in HBMVECs. Western blot results in Supplementary Figure 2areveals the successful knockdown of CREB. As expected, we found that knockdown of CREB abolished the beneficial effects of Valsartan on the expressions of VE-cadherin and Claudin 2, endothelial hyperpermeability, and TEER against high glucose (supplementary Figure 2b-d). These findings confirm the involvement of CREB in mediating the protective effects of Valsartan.

## 4 Discussion

Since high activation of AT1R in the brain plays a detrimental role in the pathology of T2D-related complications, AT1R antagonists (ARB) have been tested for neuroprotective effects in brain disorders [10]. In this study, we tested the commonly prescribed ARB compound Valsartan in diabetic mice. Diabetic db/db mice are lepr-deficient animals, which show early development of obesity, insulin resistance, and diabetes in late-stage [19]. A recent study suggests that db/db mice have significant BBB impairments at a young age [20]. AT1R is also required for leptin-sensitized metabolic control [21]; therefore, this mice line could be a very useful model to study diabetes-related brain disorders. Our study showed that AT1R in db/db mice is much higher than that in db/+ mice, suggesting that incomplete leptin signaling elevates AT1R. Therefore, antagonizing AT1R could be an effective pathway to rescue the impairment from brain disorders in db/db mice.

In our animal experiments, the administration of Valsartan for 2-weeks showed clear amelioration of BBB leakage in db/db mice, suggesting that blockage of elevated AT1R signaling may have neurovascular protection. Examination of the gap junction proteins VE-cadherin and Claudin 2 further confirmed the assumption that AT1R blockage has a protective effect on blood–brain barrier function. Based on this observation, we hypothesized that the AT1R antagonist Valsartan directly affects the integrity of neurovasculature in the brain. To investigate the direct effect of vascular integrity, we used isolated brain microvascular cells in our *in vitro* experiment. Brain vascular cell dysfunction has been established *in vitro* to mimic the hyperglycemia phenotype. Monolayer culture system studies have reported that 30 mM glucose disrupts the expression of tight junction proteins [22]. Accordingly, it induces the loss of trans-endothelial electrical resistance (TEER) [23,24]. In our experiment on cultured HBMVECs, the presence of Valsartan significantly ameliorated high glucose-induced dextran uptake and loss of TEER, suggesting it has a protective role on stress-induced hyperpermeability. Meanwhile, Valsartan also suppressed glucose stress-induced pro-inflammatory induction of IL-6 and MCP-1. As brain endothelium is an innate barrier to neuroinflammation, control of neuroinflammation can improve neurovascular function. Therefore, the amelioration of Valsartan in brain endothelial cells could involve both inflammation and neurovascular integrity. We also confirmed that Valsartan alleviated the reduction of VE-cadherin and Claudin 2, two important gap junction proteins both *in vivo* and *in vitro*. Mechanistically, we found that cellular CREB signaling is required for the action of Valsartan. In vascular cells, G protein-coupled receptor ligands induce CREB activity by mediating CREB phosphorylation at the serine 133 residue, and CREB signaling can be activated by inflammation or cellular oxidative stress. Therefore, CREB plays a critical role in vascular regulation [25]. In monolayer endothelial cells, activation of CREB prevents endothelial permeability increase [26,27]. Our results confirm that Valsartan effectively prevented the inactivation of CREB from high glucose stress. Moreover, the blockage of CREB signaling by the PKA inhibitor H89 completely abolished the effect of Valsartan, indicating that CREB activation is required for the action of Valsartan. H89 inhibits PKA by targeting its ATP-binding pocket on the C subunit, which is the catalytic component of PKA complex. The activated PKA C subunits transport into the nucleus and phosphorylate the cAMP-response element-binding (CREB) protein [28]. Therefore, H89-mediated CREB antagonism is an indirect effect. H89 has been used to inhibit the CREB pathway in the high glucose-impaired CREB pathway in a previous study [29].

The limitations of this study have to be discussed. Although we show that Valsartan exhibited neuroprotection on the integrity of the BBB in db/db mice, the therapeutic application of Valsartan in brain disorders remains to be defined. Valsartan is known to be able to penetrate the BBB [10], but its action in the brain may be AT1R-dependent or independent. We show that CREB is required for the action of Valsartan in high glucose-stressed cells, but it is still unclear how Valsartan influences the activity of CREB. Its amelioration on the activation of CREB likely requires other mediators and activates many downstream signals. In addition, the indispensable role of CREB remains to be validated in Valsartan-treated db/+ mice. We hypothesized that the blockage of CREB signaling by the PKA inhibitor H89 would diminish or abolish the therapeutic effect of Valsartan; however, an *in vivo* experiment with the Valsartan plus H89 investigation would be critical to verify the involvement of the CREB pathway. A better understanding of the molecular mechanism is essential for Valsartan’s therapeutic application. Recent studies have shown compelling data. Two separate studies demonstrate that Valsartan administration potentiates the antioxidant system and improves neuronal damages in an Alzheimer’s disease model [30,31]. In human studies, although several clinical studies report that Valsartan has a beneficial role in dementia conditions, the effectiveness of Valsartan remains to be confirmed [11,12,32,33]. These data could be the beginning of clinical trials of Valsartan in human subjects.

## Conclusion

In conclusion, our study provides both *in vivo* and *in vitro* evidence that the ARB drug Valsartan has a neurovascular protective effect in diabetic conditions. Valsartan works to ameliorate diabetes-induced brain hyperpermeability and blood–brain barrier integrity. Mechanistically, Valsartan acts to mitigate vascular inflammation and protect vascular integrity via CREB signaling in brain vascular endothelial cells. The AT1R antagonist Valsartan could play a potential role in the treatment of neurovascular complications in diabetes.

## Data Availability

Data are available upon reasonable request from the corresponding author.
